# Serum Levels of OX40 in Early and Late-Stage Oral Squamous Cell Carcinoma

**DOI:** 10.7759/cureus.14597

**Published:** 2021-04-20

**Authors:** Aliya I Sani, Zil E Rubab, Shumaila Usman, Syed Zaryab Ahmed, Mervyn Hosein, Moazzam A Shahid

**Affiliations:** 1 Biochemistry, Ziauddin University, Karachi, PAK; 2 Research, Ziauddin University, Karachi, PAK; 3 Dentistry, Ziauddin University, Karachi, PAK

**Keywords:** cancer, immune checkpoints, immunotherapy, oral squamous cell carcinoma, ox40

## Abstract

Background

The tumor necrosis factor receptor superfamily, member 4 (OX40) and its ligand (OX40L) are members of the tumor necrosis factor superfamily and play roles as costimulatory immunomodulators to combat infectious diseases as well as cancers. Presently, many therapeutic agents focused on OX40 and OX40L are in trials for antitumor efficacy. In Pakistan, oral squamous cell carcinoma (OSCC) is the second most prevalent cancer with a mortality of 50% despite the availability of various therapeutic modalities. Data regarding serum levels of OX40 in patients with OSCC is lacking. Therefore, the study aimed to assess the OX40 levels in serum and their association with the clinicopathological features of the tumor.

Methodology

A cross-sectional study was conducted and serum samples of 78 biopsy-confirmed OSCC patients were collected prior to any treatment along with 10 healthy persons after informed consent. Serum levels of OX40 were measured via sandwich enzyme-linked immunosorbent assay (ELISA).

Results

The mean serum levels of OX40 were 1.65 ± 0.64 ng/ml and 2.39 ± 0.58 ng/ml in early and late-stage disease patients of OSCC, respectively (*p *=<0.005). However, based on gender and tumor site, male gender and buccal mucosa tumors in late-stage OSCC patients showed higher mean levels of OX40, 2.42± 0.58 ng/ml and 2.41 ± 0.58 ng/ml (*p *=<0.05), respectively. Patients with well-differentiated tumors demonstrated mean serum levels of 2.28 ng/ml, and in moderately differentiated tumors, the mean levels were 2.19 ng/ml (*p *=0.47).

Conclusions

A high OX40 level is associated with advanced-stage disease and a poor prognosis, possibly reflecting the immune-exhausted status against OSCC.

## Introduction

Oral squamous cell carcinoma (OSCC) remains one of the most prevalent cancers, with the incidence in 2020 being about 377,713 and with reports of 177,757 deaths worldwide due to it [1]. OSCC is a subset of head and neck squamous cell carcinoma; it includes squamous cell carcinomas of the lip, tongue, and oral cavity [2]. The best ways to prevent OSCC are abstinence from smoking, alcohol, and chewable tobacco products. Another risk factor that is attributable to OSCC is HPV infection [3]. In Pakistan, OSCC is the most common malignancy reported in males with twice the incidence as compared to females [1]. It affects not only elderly, aged people but also youth due to the abundant consumption of smokeless tobacco in this region. Despite the availability of different treatment options, the five-year survival rate has remained 50%. One of the main reasons for this lies in the fact that OSCC is often difficult to diagnose until cancer has advanced [4]. Oral cancer may be cured by either surgery or radiation [5]. Furthermore, invasive surgical techniques may result in reduced quality of life. To date, there have not been any established protocols or diagnostic tools for the early detection of OSCC. Currently, the standard of care is routine oral cancer screenings in at-risk populations; despite that, such screening tools are far from early diagnosis [6].

Presently, research is being undertaken on the early diagnosis and prevention of different cancers in order to improve outcomes. The research focuses on “Biological Hallmarks of Cancer” and one of them is an evasion of immunity [7]. The newer immunotherapeutic agents are being evaluated in trials to boost antitumor activity in form of monotherapy and a combination of other treatment options in various cancers, including tumors of the head and neck. The immune mediators that are under research are programmed cell death protein 1 (PD1), programmed death-ligand 1 (PDL1), T-cell immunoglobulin domain and mucin domain (TIM), and tumor necrosis factor receptor superfamily, member 4 (OX40) and its ligand (OX40L), but the trials have shown beneficial effects, however, at the cost of side effects and the development of resistance to therapy [8].

"Tumor necrosis factor receptor superfamily, member 4" (OX40, CD134) and its ligand “tumor necrosis factor (ligand) superfamily, member 4” (OX40L, CD252) are immune costimulatory molecules. Interest in them has increased over the last two decades so that therapeutic agents to boost anti-tumor immunity can be devised. OX40 is fundamental in augmenting T-cell responses initiated by the T-cell receptor (TCR). Its expression is reported in T cells and antigen-presenting cells (APCs). Studies based on the expression and serum levels of OX40 have shown a variable association with prognosis in different cancers [9]. Data regarding the serum levels of OX40 in patients with OSCC is lacking. These markers are currently being tested to predict the biological behavior and prognosis of different other cancers. The current study assessed the potential role of serum OX40 levels in OSCC and its relation with the clinical stages and histological grades of OSCC.

## Materials and methods

A cross-sectional study was designed to measure and assess the serum levels of OX40. Ziauddin University's ethics committee approved this research proposal (ERC#2410720ASBC). Serum samples were obtained from 78 patients with histologically confirmed OSCC patients prior to any treatment and from 10 healthy subjects after written informed consent. Samples were collected from October 2020 till February 2021. Patients with a history of cancers in other parts of the body, systemic disease, chemotherapy, or radiotherapy were excluded from the study. The demographic and clinicopathological data were collected from patients and their medical documents such as CT scans and biopsy reports.

Blood sampling

Peripheral venous blood (3 ml) was collected by undertaking aseptic measures by a trained phlebotomist. Samples were then transferred in yellow-topped serum collection bottles. The collected blood samples were centrifuged for 10 min at 1500 rpm to separate serum and other cellular elements. The serum was then decanted and aliquots were made and stored in a -80°C freezer till further analysis.

Enzyme-linked immunosorbent assay (ELISA)

The levels of OX40 were measured by sandwich ELISA, following the manufacturer’s protocol (SEB519Hu; Cloud-Clone Corp, TX). In each well of a 96-well plate, 100 µl of serum was added and incubated for one hour at 37°C followed by the removal of the fluid from the wells. Subsequently, 100 µl of detection reagent A was added to each well and incubated for one hour at 37°C, aspiration of the solution was performed, and in the successive step, three-time washing of the wells was done by wash solution. After that,100 µl of detection reagent B was added to each well and incubated for 30 minutes at 37°C. After aspiration, the well was washed five times. In the next step, 90 µl of substrate solution was added for 10-20 minutes, followed by 50 µl of stop solution, to each well. Then, the ELISA plate was read at 450 nm in the MultiSkan Spectrophotometer (Thermo Fisher Scientific, Waltham, Massachusetts).

Statistical analysis

The Statistical Package for the Social Sciences (SPSS) version 25.0 (IBM Corp., Armonk, NY) was employed for data analysis. An independent sample t-test was used to compare mean OX40 serum levels between the different stages and grades of oral SCC patients. The OSCC patients were categorized according to the clinical stages into early staged (Stage I and II) and late staged (Stage III and IV) patients. The *p *value of < 0.05 was considered statistically significant.

## Results

A total of 78 biopsy-proven cases of oral squamous cell carcinoma prior to any treatment were included in this study along with 10 healthy individuals. The mean age of healthy subjects (n=10) was 42±5.8 ng/ml while the ages of patients in the early stages and late stages were 46±13 ng/ml and 49.7±13.06 ng/ml, respectively. Among the OSCC patients, male gender (n=68;86%) showed a higher predilection of OSCC as compared to females (n=11;14%) with a male to female ratio of 6:1. The common site of tumor reported in our samples was the buccal mucosa (n=56; 71.8%) followed by the lip (n=13; 16.7%), tongue (5; 6.4%), and palate (n=4; 5.1%) although the most common habit was the consumption of paan with smoking (32%) followed by smoking alone (19.2%).

**Figure 1:**
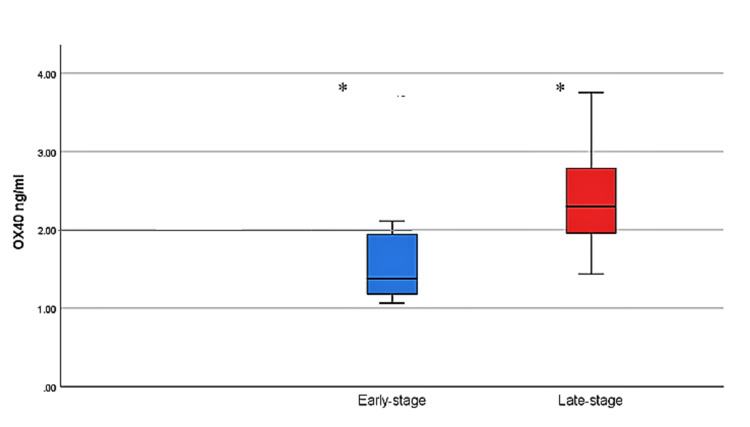
Means levels of OX40 in early and late-stage OSCC patients OX40: tumor necrosis factor receptor superfamily, member 4; OSCC: oral squamous cell carcinoma

OSCC patients were categorized according to the clinical stages into early staged (Stage I and II) and late staged (Stage III and IV) patients. The mean OX40 levels in healthy individuals (n=10) was 1.98 ± 0.50 ng/ml, whereas in early staged and late staged patients, mean serum levels of 1.65 ± 0.64 ng/ml and 2.39 ± 0.58 ng/ml were observed (*p*=<0.005), respectively (Figure 1).

The mean OX40 levels in early and late-stage cancer patients with age, gender, site, and habit are depicted in Table 1. 

**Table 1:** Mean OX40 serum levels in demographic variables of OSCC patients OX40: tumor necrosis factor receptor superfamily, member 4; OSCC: oral squamous cell carcinoma

Variables	OSCC patients (mean ±SD) ng/ml		p-value
	Early Stage	Late Stage	
Age			
< 50 years	1.68±0.75**	2.26±0.47**	0.017*
>50 years	1.61±0.45**	2.49±0.67**	0.005*
Gender			
Male	1.49 ± 0.39**	2.42 ± 0.58**	<0.005*
Female	2.63 ± 1.28	2.17 ± 0.57	0.44
Site of tumor			
Buccal mucosa	1.48±0.39	2.41±0.58	0.00*
Lip	1.96 ±0.95	2.43±0.6	0.36
Habit			
Paan	1.24	2.45±0.69	0.132
Smoking	2.0±0.97	2.45±0.55	0.375
Paan and smoking	1.70±0.32	2.31±0.56	0.009*

According to histological grading, well-differentiated squamous carcinoma was the most frequent grade observed (n=45;57.6%) in our study samples followed by moderately differentiated (n=32: 41%). However, poorly differentiated squamous cell carcinoma was reported in only one sample (1.28%). The mean serum levels of OX40 in well-differentiated OSCC was 2.28 ng/ml and it was 2.19 ng/ml in moderately differentiated OSCC, with no significant difference among the grades (*p*=0.47).

## Discussion

Tumor necrosis factor receptor 4 (OX40) is a member of the tumor necrosis factor superfamily and mainly functions as the costimulatory immune molecule. The expression is reported in activated T cells as well as in other immune cells [10]. It is thought to be the potential target for enhancing antitumor immunity, hence presently, multiple pharmaceutical agent trials focusing on OX40 agonists are in process. The current study demonstrated that the serum OX40 levels in patients with late-stage OSCC were higher as compared to early staged patients (p=<0.005). Blood samples can be collected non-invasively giving an edge on tumor biopsy samples, as blood bespeaks not only the patient’s body status but also the milieu of the whole tumor tissue [11]. According to our literature search, no reports relating to serum OX40 levels in OSCC and its relationship with different histological grades and clinical stages were found.

The cornerstone of cancer treatment is fundamentally based on staging and grading. A few studies have been conducted on the serum levels of OX40 in different malignancies. Adult patients of T-cell leukemia demonstrated elevated serum OX40 levels while Sawada et al. reported an association between higher serum levels of OX40 and reduced survival time in advanced stage colorectal patients (n=22) [12-13]. Similarly, Kashima et al. in advanced lung adenocarcinoma (n=56) reported higher levels were associated with poor prognosis [11]. These higher serum levels may reflect immune exhausted status against the cancers in advanced stages.

Many studies have been conducted to evaluate the expression of OX40 in tumor-infiltrating lymphocytes (TILs) and the association of OX40 with prognosis has varied across different types of cancers. Xie et al. discovered that in hepatocellular carcinoma, the higher OX40 expression in TILs correlated with poor survival [14]. Similarly, in myeloid leukemia, shorter survival was related to OX40 expression while in patients with an early stage of “non-small cell lung carcinoma” (NSCLC), higher TIL OX40L expression had a poor prognosis [15-16]. On the contrary, lower OX40 expression was correlated with chemosensitivity as well as recurrence-free survival in ovarian cancer patients.

There are also contrasting results reported in head and neck squamous cell carcinoma (HNSCC). A study by Bell et al. quoted in advanced stage HNSCC abundance of OX40 expressing T-cells was observed in the tumor tissues [17]. Present study findings are also in accordance with Bell et al.'s findings reflected by higher serum levels in advanced stages. The fact that the inflammation at the tumor site and in the body may affect serum levels. Additionally, OX40 expression on the CD4+ T cell surface was significantly decreased in patients with advanced tumors in comparison to early staged patients [18]. In OSCC, the abundance of regulatory T-cells (Tregs) in TILs is associated with poor survival, the expression of OX40 is reported to be significantly greater in the sub-population of Treg in TILs comparatively to CD4 and CD8 population of TILs and to the Treg isolated from peripheral blood lymphocytes [19-20]. The notion of a stimulating immune system against malignancies led to the exploration of different immunotherapeutic agents and different clinical trials have demonstrated the efficacy of immune modulators. Oberst et al. demonstrated that the human OX40L IgG4P Fc fusion protein (MEDI6383) subdues the Tregs function of immunosuppression by inducing the activation of T-cells in vitro and in vivo models [21]. The current trial of OX40 agonist demonstrated an increase in TILs and improvement in the patients with an adequate safety profile [22]. However, the effect of immune checkpoint blockade therapy on OSCC is limited and the subtypes within the TILs and expression of immune cell subtypes should be also taken into account [23].

The sites affected by oral cavity cancers vary in different geographical regions due to the nature of exposure and genetic history. In this study, the majority of the patients had carcinoma in the buccal mucosa (71%) followed by lip (16%). A study from southern Punjab reported tumors of the tongue (50%) as the most frequent. Another study carried out in Pakistan showed that tumors of buccal mucosa were the most frequent (32%), followed by tumors of the tongue (22%). The reason for that might be that the use of smokeless tobacco is a culturally acceptable habit in the forms of gutka, areca nut, paan, and naswar, which tends to be rising [24]. People tend to keep smokeless tobacco products lodged in the buccal mucosa for prolonged times, from where it releases its juices slowly and aids in the carcinogenic effect of these products.

The measurement of OX40 in the serum of OSCC patients along with its expression evaluation at mRNA level in blood and saliva may shed light on new immunotherapies against OSCC. In the future, longitudinal and larger sample studies would reveal the value of OX40 as a predictive biomarker for therapy in OSCC patients. We acknowledge that the small sample size of 78 OSCC patients was the main limitation of this study.

## Conclusions

Based on the findings, the present study revealed that a high OX40 level is associated with advanced-stage disease and a poor prognosis possibly reflecting the immune-exhausted status against OSCC, especially in the male gender, and buccal mucosa tumors. Nevertheless, the state of inflammation at different tumor sites may impact OX40 levels. These findings suggest that serum OX40 levels represent a controlling role rather than having a provocative effect on immunity against tumors and can predict the efficacy of such agents. Serum OX40 levels may reflect the severity of disease in patients with oral squamous cell carcinoma and may aid in the rationalizing for investigating serum levels and prescribing anti-OX40 agonists in such patients in the future.
